# Synaptic Dysregulation Drives Hyperexcitability in Pyramidal Neurons Surrounding Freeze-Induced Neocortical Malformations in Rats

**DOI:** 10.3390/ijms26041423

**Published:** 2025-02-08

**Authors:** Sergey L. Malkin, Dmitry V. Amakhin, Elena B. Soboleva, Tatiana Y. Postnikova, Aleksey V. Zaitsev

**Affiliations:** Laboratory of Molecular Mechanisms of Neural Interactions, Sechenov Institute of Evolutionary Physiology and Biochemistry, Russian Academy of Sciences, 194223 Saint Petersburg, Russia; adresatt@gmail.com (S.L.M.); dmitry.amakhin@gmail.com (D.V.A.); soboleva.elena.1707@gmail.com (E.B.S.); tapost2@mail.ru (T.Y.P.)

**Keywords:** epileptiform activity, focal cortical dysplasia, microgyria, freeze lesion rat model, synaptic transmission, excitation–inhibition balance, neocortex, drug-resistant epilepsy, seizure susceptibility, pyramidal neurons

## Abstract

Focal cortical dysplasia (FCD) is a leading cause of drug-resistant epilepsy; however, the mechanisms underlying hyperexcitability in the affected cortical regions remain poorly understood. In this study, we employed a freeze-induced neocortical malformation model in rats to investigate the electrophysiological properties of pyramidal neurons surrounding the microgyrus and to evaluate changes in synaptic transmission. Using whole-cell patch-clamp recordings, we analyzed passive and active membrane properties, synaptic responses, and epileptiform activity in brain slices from rats with FCD and sham-operated controls. Our results revealed that while the intrinsic biophysical properties of neurons remained largely unchanged, the summation of excitatory and inhibitory inputs was significantly enhanced. Notably, the balance of inhibitory and excitatory synaptic currents was shifted toward excitation, making the perilesional cortex more susceptible to seizure generation. In a model of epileptiform activity induced by GABA_A_ receptor blockade and reduced Mg^2+^ concentration, we observed early ictal activity originating in the microgyrus and spreading to adjacent regions. These findings demonstrate that synaptic perturbations, rather than alterations in intrinsic neuronal properties, are the primary drivers of hyperexcitability in this model. Our study highlights the importance of synaptic dysregulation in FCD-related epilepsy and suggests that targeting synaptic transmission may offer a promising therapeutic strategy for controlling seizures in patients with cortical malformations.

## 1. Introduction

Epilepsy is a severe chronic neurological disorder, clinically characterized by a predisposition to recurrent uncontrolled seizures. The most severe forms of epilepsy, which are poorly treatable and manifest from early childhood, result from congenital structural anomalies of the cerebral cortex [1,2,3]. Neocortical malformations represent a significant and diverse group of neuronal abnormalities. These malformations, often associated with epilepsy, include structural changes such as altered cortical thickness (e.g., microcephaly, macrocephaly), abnormal folding patterns (e.g., lissencephaly, polymicrogyria, dysgyria), disrupted lamination (e.g., focal cortical dysplasia), and the presence of ectopic cell clusters (e.g., heterotopia) [3].

It has been demonstrated that chemical or physical manipulation of pre- and neonatal animals can result in the development of neocortical malformations that bear resemblance to various human pathologies [4]. In this study, we employ the freezing lesion model of focal cortical dysplasia (FCD) as proposed by Dvorak et al. [5,6]. In one-day-old rats, rapid local freezing of the cortex causes necrosis in the infragranular layers. This leads to the migration of supragranular neurons through the damaged area. As a result, a microgyrus forms, consisting of a three- to four-layer cortex. This includes a molecular layer, a thickened layer II, a dissecting lamina (which replaces necrotic layers IV, V, and VI), and a layer IV of neurons that correspond to layer VI in the intact cortex [1,6].

It has been shown previously that neocortex slices obtained from juvenile and adult rats that received focal freezing shortly after birth exhibit marked excitability in the cortical area surrounding the FCD region. The epileptiform network activity evoked by local electrical stimulation of dysplastic tissue extended several millimeters in a horizontal direction [7,8,9]. Intracellular and patch-clamp in vitro recordings from single neurons located in close proximity to the FCD area demonstrate the occurrence of multiphasic and sustained discharges evoked by extracellular stimulation, which coincide with extracellularly recorded network activity [10,11]. Furthermore, epileptiform activity has been observed to extend not only to neocortical areas in close proximity to the FCD region but also to areas situated at a relatively greater distance [12].

The neuronal and synaptic mechanisms of ictogenesis in this model remain poorly understood. It is hypothesized that both the membrane properties of neurons in the lesion area and the balance between glutamatergic and GABAergic systems are disrupted. For example, previous studies employing a model of FCD with focal freezing have demonstrated an imbalance in the binding of excitatory and inhibitory mediators to their respective receptors. For example, increased binding of kainate to AMPA receptors and reduced binding of GABA to GABA_A_ receptors have been observed. These changes may contribute to the hyperexcitability seen in this model [13]. Additionally, it was demonstrated that the number of GABAergic neurons was diminished by a factor of two to three in the region of the lesion [10]. Additionally, the frequency of spontaneous and miniature EPSPs was higher, suggesting an increase in excitatory synaptic inputs. This may result from hyperinnervation of thalamocortical or intracortical pathways [14,15].

The aim of this study is to examine the electrophysiological properties of pyramidal neurons in layers 2–3 near the microgyrus, along with their excitatory and inhibitory synaptic inputs.

## 2. Results

### 2.1. Morphology

**Figure 1:**
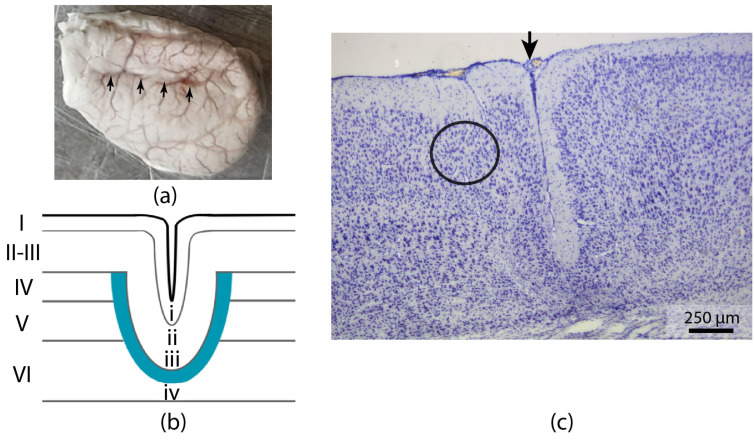
Freeze-induced neocortical microgyria. (**a**) Representative example of a rat brain (P21) that sustained freeze-induced damage at age P0, resulting in a microgyrus in the left sensorimotor cortex (shown with arrows). (**b**) Schematic representation of the freeze-induced microgyria. The Roman numerals represent the cortical layers. (**c**) Representative example of a Nissl-stained coronal section through a cortical lesion in the rat cerebral cortex. The image clearly shows a formed four-layered microgyrus. The circle indicates the area where neuronal activity was recorded. The black arrow marks the microgyrus.

The neocortical microgyria in the left cortical S1 area was induced by forming a freeze lesion ≈ 2 mm posterior to the bregma and ≈2 mm lateral to the midline, with three additional lesions ≈ 1 mm apart in a rostro–caudal direction. The formation of microgyri was analyzed in rats aged 21 days. The results demonstrated that a focal freeze lesion resulted in the formation of an approximately 8 mm long microgyrus in the region of the somatosensory cortex (Figure 1a). Nissl-stained sections demonstrate a microgyrus with a three- to four-layered cortex, comprising a molecular layer, a thickened layer II, a dissecting lamina (corresponding to necrotic layers IV, V, and partially VI), and layer IV of neurons corresponding to layer VI of the intact cortex (Figure 1b,c).

### 2.2. Membrane Properties

#### 2.2.1. Passive Membrane Properties

**Figure 2:**
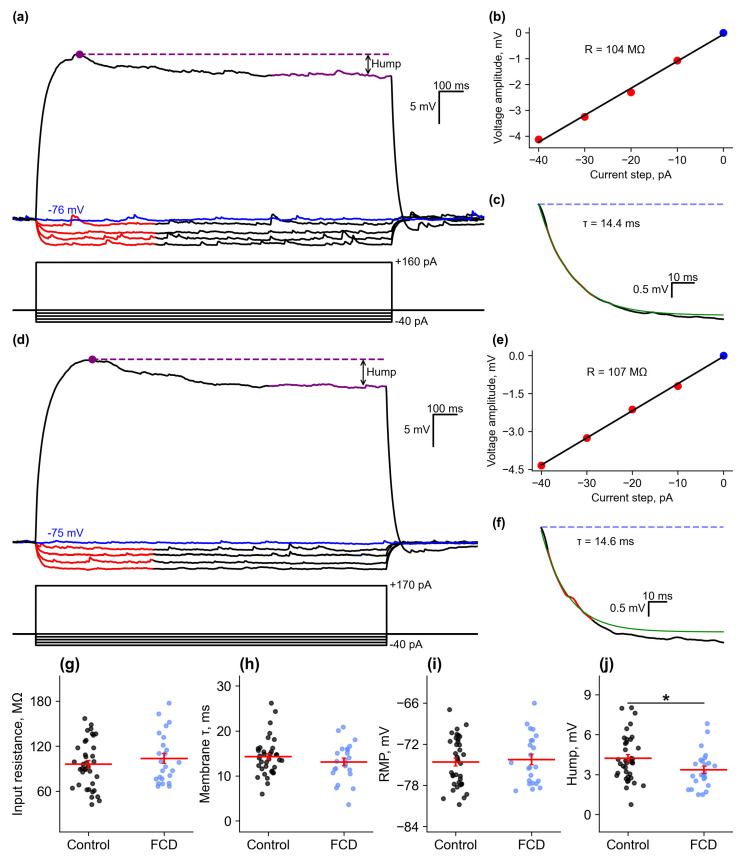
Passive membrane properties. (**a**) Example of neuron responses to hyperpolarizing and subthreshold depolarizing current steps, control. The voltage trace utilized for assessing the RMP is marked with blue. The recording fragments used for the input resistance calculation are marked in red. (**b**) Illustration of calculating a neuron’s input resistance by linear approximation of its current–voltage characteristic. The red and blue dots represent the values obtained from the parts of the recording from (**a**) marked with the respective color. (**c**) Illustration of calculating the membrane time constant using an exponential approximation (green) of the neuronal response to a depolarizing current step. (**d**–**f**) The same for a neuron in FCD group. (**g**–**j**) Plots showing values of input resistance, membrane time constant, resting membrane potential (RMP), and depolarization-activated current amplitude (hump) for neurons in control animals (sham) and neurons near the microgyrus region in rats with the FCD model. * *p* < 0.05, Student’s *t*-test.

Recordings were made in pyramidal neurons of layer II-III of the cortex, located near the microgyrus approximately 50–300 µm from the lesion center, in a normally formed six-layer cortex (Figure 1c). The RMP was not corrected for LJP and did not differ between groups (control: −74.6 ± 0.6 mV, *n* = 37; FCD: −74.2 ± 0.8 mV, *n* = 23). Input resistance (R_in_) and membrane time constant (τ) were measured in response to the application of a hyperpolarizing current; these parameters also did not differ between groups (R_in_: control: 95.9 ± 5.2 MΩ, *n* = 37; FCD: 103.8 ± 6.7 MΩ, *n* = 24; τ: control: 14.3 ± 0.7 ms, *n* = 36; FCD: 13.1 ± 0.9 ms, *n* = 23) (Figure 2). The hump was relatively small in both groups, being 4.24 ± 0.29 mV in the control group (*n* = 37) and 3.37 ± 0.28 mV in the FCD group (*n* = 24), *p* < 0.05.

#### 2.2.2. Action Potential Properties

**Figure 3:**
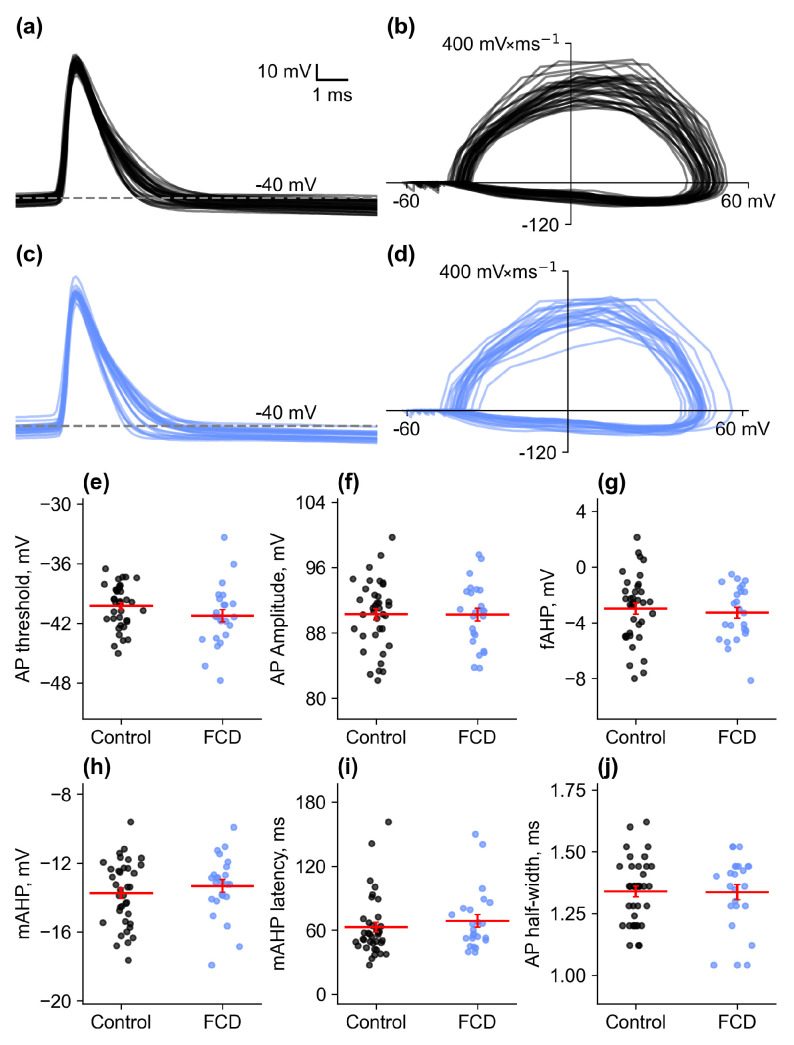
(**a**) Complete sample of APs examined from the control group. (**b**) Phase diagrams for the APs shown in (**a**). (**c**) Complete sample of APs analyzed in the FCD group. (**d**) Phase plots for the APs shown in (**c**). (**e**–**j**) Values of AP threshold and amplitude, fast and medium afterhyperpolarization amplitudes (fAHP and mAHP), mAHP latency, and AP half-width for neurons in control animals and neurons near the microgyrus region in rats with the FCD model.

The characteristics of the action potentials were analyzed at the level of the rheobase current. Figure 3 shows the action potentials recorded in the control and FCD groups, and phase diagrams for these action potentials are also shown. The action potential characteristics in the control and FCD groups were similar, with no observed differences in threshold size (control: −40.3 ± 0.4 mV, *n* = 37; FCD: −41.3 ± 0.6 mV, *n* = 24), amplitude (control: 90.3 ± 0.7 mV, *n* = 37; FCD: 90.2 ± 0.8 mV, *n* = 24), or AP half-width (control: 1.34 ± 0.02 ms, *n* = 37; FCD: 1.34 ± 0.03 ms, *n* = 24). Afterhyperpolarization (AHP) also did not differ between these groups. The fast AHP had a small amplitude (control: −3.0 ± 0.4 mV, *n* = 37; FCD: −3.3 ± 0.4 mV, *n* = 24), followed by a larger amplitude intermediate AHP (control: −13.8 mV ± 0.3, *n* = 37; FCD: −13.3 ± 0.4 mV, *n* = 24) with a latency of approximately 65 ms (control: 63 ± 5 ms, *n* = 37; FCD: 69 ± 6 ms, *n* = 24). Therefore, FCD did not appear to affect the basic characteristics of neuronal action potentials in adjacent areas.

#### 2.2.3. Firing Properties

**Figure 4:**
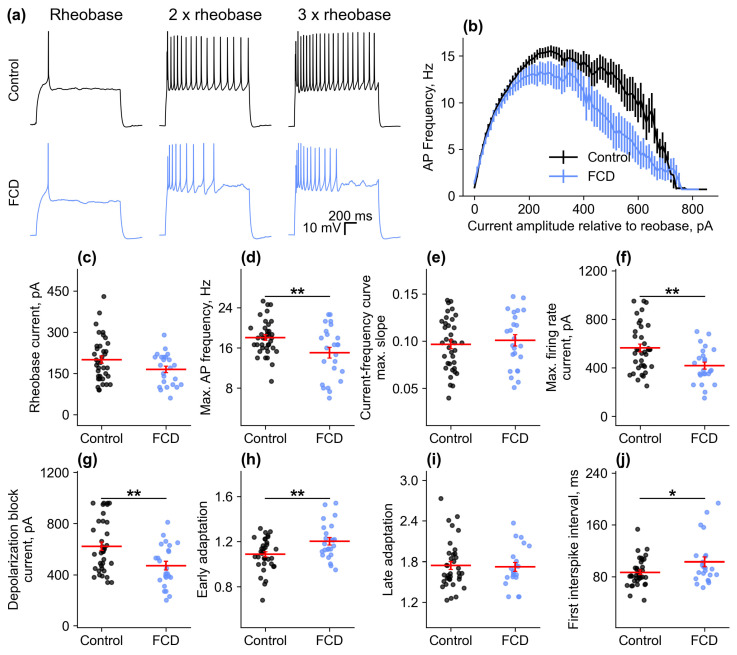
Firing pattern characteristics. (**a**) Examples of recordings of firing patterns in controls and in neurons near the microgyrus at one, two, and three rheobases. (**b**) Averaged frequency–current characteristics in controls and in neurons near the microgyrus. Current values are adjusted to rheobase current. (**c**–**j**)—Values of rheobase current, maximum firing frequency, maximum slope of the frequency–current curve, current causing maximum firing frequency, minimum current causing depolarization block, early and late firing frequency adaptation, and first interspike interval for neurons in control animals (sham) and neurons located near the microgyrus in rats with the FCD model. * *p* < 0.05, ** *p* < 0.01, Student’s *t*-test.

Next, we assessed and compared the excitability properties, including the rheobase current, current eliciting the maximal AP frequency, time of the first spike, maximal AP frequency, and maximal current–frequency curve (CFC) slope, as well as early and late frequency adaptation (Figure 4).

At lower currents (up to 200 pA above rheobase), neuronal firing frequency increased linearly with increasing injected current, and neuronal responses were similar in both groups. At higher currents, the responses of the control and FCD groups began to differ. As the current increased, neurons in the FCD group switched to depolarizing block earlier, causing the firing frequency to decrease quite rapidly as the current continued to increase. As a result, some firing characteristics significantly differed between the groups. The FCD group demonstrated lower maximal firing frequency (control: 18.1 ± 0.6 Hz, *n* = 37; FCD: 15.0 ± 1.1 Hz, *n* = 24, *p* < 0.01). The minimal current eliciting the maximal firing frequency was also lower (control: 564 ± 32 pA, *n* = 37; FCD: 418 ± 30 pA, *n* = 24, *p* < 0.01), as was the minimal current eliciting the depolarizing block (control: 622 ± 33 pA, *n* = 37; FCD: 470 ± 33 pA, *n* = 24, *p* < 0.01).

Thus, FCD did not seem to affect the main biophysical properties of neurons: passive membrane properties, as well as action potential properties of neurons in adjacent cortical areas, did not differ from those of the control group. Some changes in neuronal properties during burst activity are more indicative of compensatory changes aimed at reducing susceptibility to epileptiform activity. In particular, firing frequency decreased, and a lower depolarizing current caused firing disruptions and depolarizing block.

### 2.3. Alterations in Synaptic Properties

**Figure 5:**
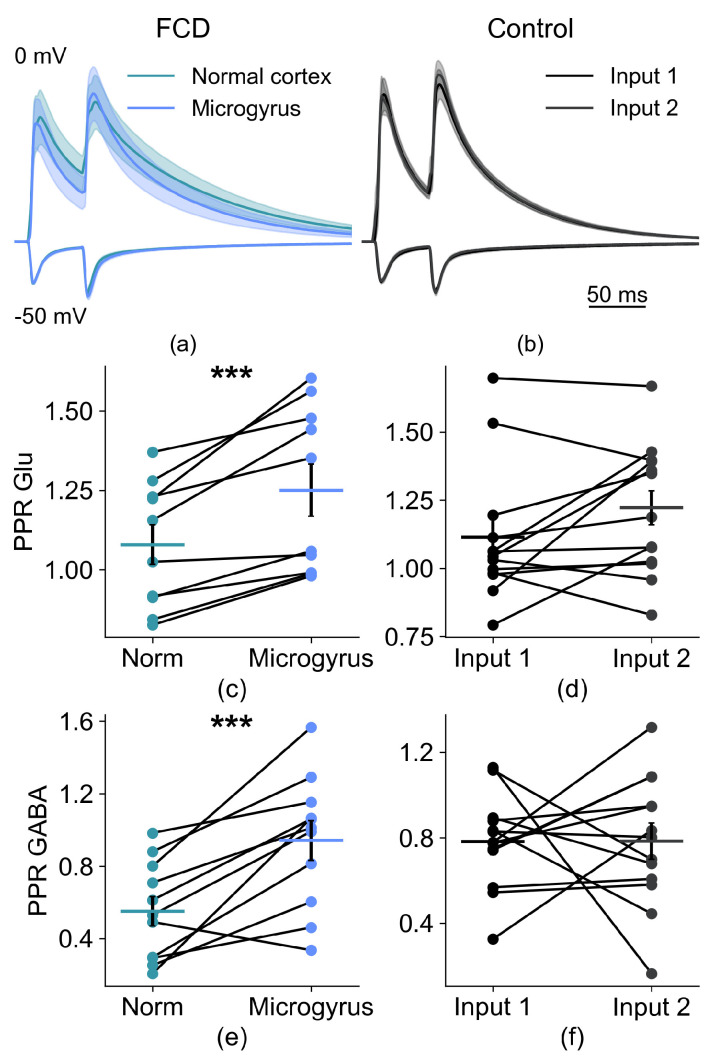
Characteristics of synaptic responses of the layer 2/3 pyramidal cells near the FCD to paired-pulse stimulation. (**a**) averaged recordings of paired PSCs in the FCD group. Bottom traces were recorded at V_hold_ = −50 mV, top at 0 mV. The stimulation was performed from two locations for each neuron, one located in the microgyrus, and the other at the same distance from the neuron in the normally formed cortex. (**b**) the same for the control group. Here, both stimulating electrodes were located in the same layer as the studied neuron, at equal distances from it. (**c**) paired-pulse ratios (PPR) in the FCD group for responses, recorded at −50 mV. (**d**) same for the control group. (**e**) paired-pulse ratios (PPR) in the FCD group for responses, recorded at 0 mV. (**f**) same for the control group. *** *p* < 0.001, Student’s *t*-test.

In the next part of our study, we analyzed the properties of the synaptic inputs to these neurons. First, we investigated whether the evoked responses differed when extracellular stimulation was applied from the FCD region and layer 2/3 of the normal cortex (Figure 5). We delivered a pair of pulses (50-millisecond interstimulus intervals) and recorded synaptic current responses at membrane potentials of −50 mV and 0 mV, a configuration that allowed, based on the different reversal potentials, for a separation of glutamatergic and GABAergic currents, respectively. In animals that underwent sham surgery, no microgyrus was formed. Therefore, we performed simultaneous extracellular stimulation in a similar cortical region with one stimulating electrode placed 50–300 µm to the right of the targeted neuron, and the other—at the same distance to the left of it.

We found that in animals with FCD, the paired-pulse ratio (PPR) of synaptic current amplitudes from microgyrus stimulation was higher than that from normal cortex for both glutamatergic (normal cortex: 1.08 ± 0.06; FCD: 1.25 ± 0.08; *n* = 12; *p* < 0.001) and GABAergic (normal cortex: 0.55 ± 0.08; FCD: 0.94 ± 0.11; *n* = 12; *p* < 0.001) currents. As expected, in sham-operated animals, no differences were found between responses from left and right stimulation (glutamatergic EPSCs: 1.11 ± 0.06 vs. 1.22 ± 0.06, *n* = 15; *p* > 0.05); GABAergic EPSCs: 0.78 ± 0.06 vs. 0.78 ± 0.08, *n* = 15; *p* > 0.05).

**Figure 6:**
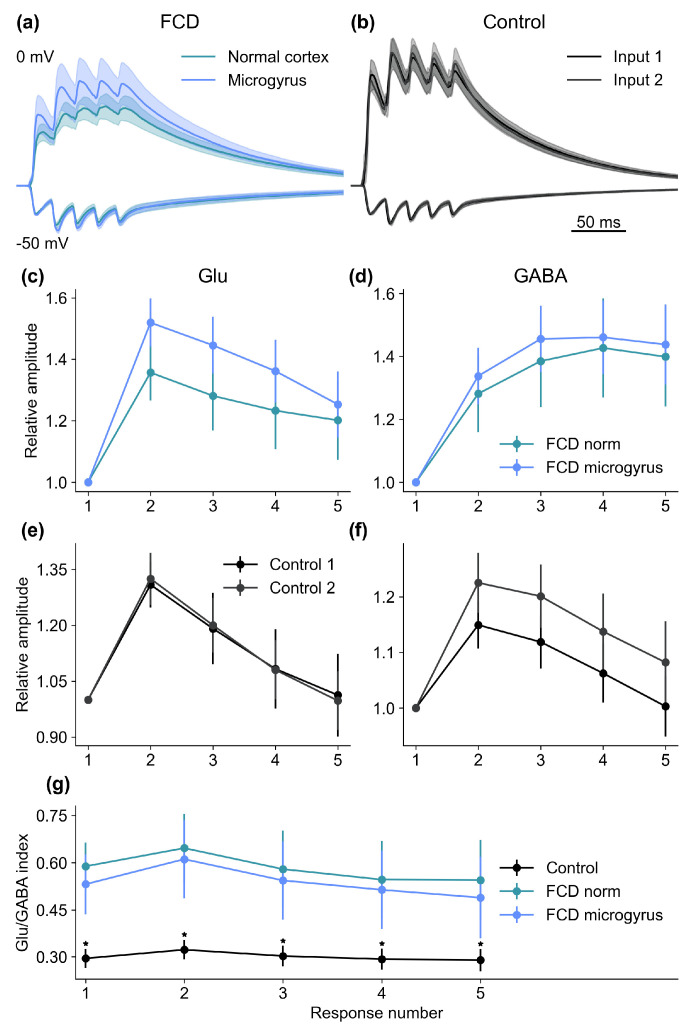
Characteristics of synaptic responses of the layer 2/3 pyramidal cells near the FCD to train stimulation. (**a**) averaged recordings of paired EPSCs in the FCD group. Bottom traces were recorded at V_hold_ = −50 mV, top at 0 mV. The stimulation was performed from two locations for each neuron, one located in the microgyrus, and the other at the same distance from the neuron in the normally formed cortex. (**b**) the same for the control group. Here, both stimulating electrodes were located in the same layer as the studied neuron, at equal distances from it. (**c**) summation curves in the FCD group for responses, recorded at −50 mV. All amplitudes are normalized to the amplitude of the first EPSC in the train. (**d**) same for the responses recorded at 0 mV. (**e**) summation curves in the control group for responses, recorded at −50 mV. All amplitudes are normalized to the amplitude of the first EPSC in the train. (**f**) same for the responses recorded at 0 mV. (**g**) Ratio of the amplitudes of glutamatergic EPSCs recorded at −50 mV to amplitudes of GABAergic ISPCs recorded at 0 mV (Glu/GABA index) for control and FCD groups. * *p* < 0.001, Tukey post hoc test.

Next, we decided to compare the summation of synaptic inputs from the FCD region with the input from the normal cortex. For these purposes, the stimulation electrode positions and recording conditions were the same as in the paired-pulse experiments described above. However, the stimuli were delivered in trains of five at 50 Hz. The recorded current amplitudes were then normalized to the peak amplitude of the first EPSC in the train, and summation curves were plotted and analyzed for each neuron (Figure 6).

In animals with FCD, the summation curves were similar for the synaptic inputs from the FCD region and from the normal cortex for both glutamatergic (F_1,60_ = 0.320, *p* > 0.05) and GABAergic responses (F_1,60_ = 0.012, *p* > 0.05). We then proceeded to analyze the ratio of the glutamatergic and GABAergic response amplitudes in the train (Glu/GABA index) for these neurons and compared it to the ratio observed in the control group. Our findings revealed that the Glu/GABA index in the FCD group was approximately 85% higher than in the control group (F_2,119_ = 14.55, *p* < 0.001). This difference was observed for both the input from the FCD region (FCD: 0.538 ± 0.051; control: 0.300 ± 0.014; *p* < 0.001, Tukey post hoc test) and from the normal cortex (normal cortex: 0.581 ± 0.048; control: 0.300 ± 0.014; *p* < 0.001). Therefore, we observed a shift in the inhibition–excitation balance toward excitation in neurons adjacent to the FCD microgyrus.

### 2.4. Characteristics of Epileptiform Activity in Animals with FCD

**Figure 7:**
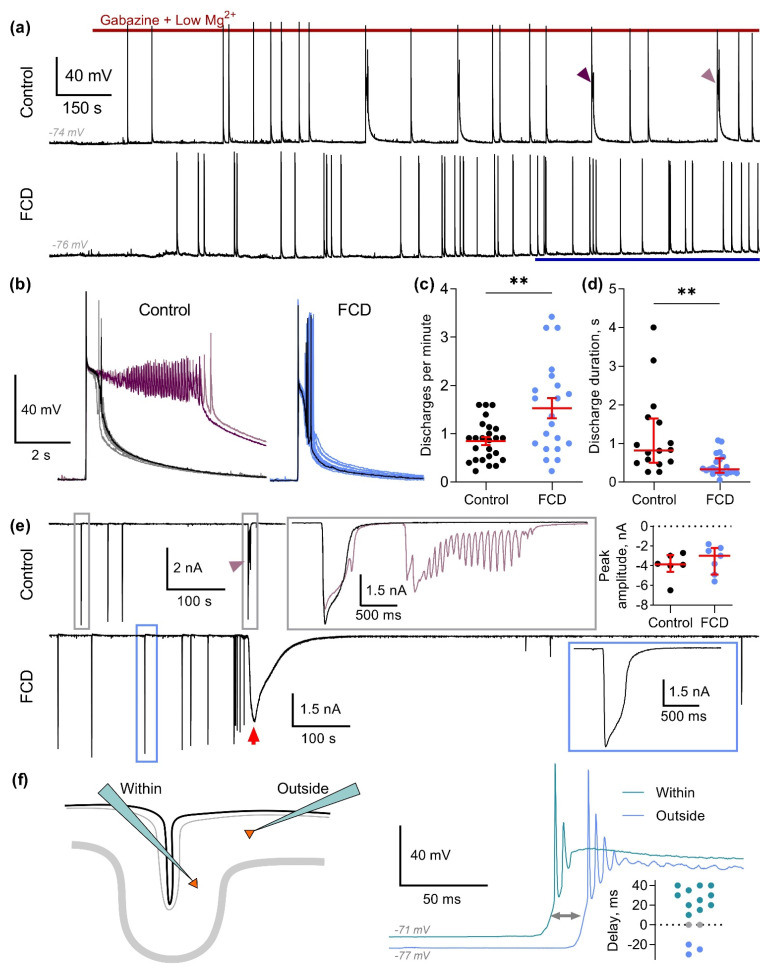
Epileptiform activity in parietal cortex slices from control rats (Control) and rats with FCD. (**a**) Representative current-clamp recordings from parietal cortex neurons during perfusion of the slice with the modified ACSF with decreased Mg^2+^ concentration and gabazine. A blue line below the recording marks the time period during which average discharge duration and frequency were assessed. Purple triangles mark the prolonged discharges. (**b**) The superposition of individual discharges from (**a**). Note that a fraction of the discharges in the control group has a significantly longer duration (marked in purple). (**c**) The frequency of epileptiform discharges is significantly higher in the FCD group (Welch’s test, ** *p* < 0.01). (**d**) The duration of epileptiform discharges is significantly lower in the FCD group (Mann–Whitney test, ** *p* < 0.01). (**e**) Representative voltage-clamp recordings (at −60 mV) of synaptic currents during the generation of epileptiform discharges. Representative discharges are extended in the gray box for control and in the blue box for FCD group. Note that some discharges in the recording from the control group had a higher duration (marked with a purple triangle and extended in purple). The diagram (top right) represents the comparison of peak amplitudes of the synaptic currents (*p* = 0.35, Mann–Whitney test). The red arrow in the bottom trace marks an episode of CSD, after which the generation of epileptiform discharges is abolished. (**f**) Dual current clamp recordings of epileptiform discharges in slices with a freeze-induced lesion. The left panel shows the relative position of the two recorded neurons and the lesion. The right panel shows the superimposed recordings of epileptiform discharge initiation. Note that discharge onset was detected 20 ms earlier in the neuron inside the lesion than in the neurons outside the lesion. The distribution of the delays between the discharges, recording within and outside the microgyrus, is plotted in the bottom right corner. Green dots (positive values) represent the recordings in which the discharges were first detected within the microgyrus; blue dots (negative values) represent the recordings in which the discharges were first detected outside the microgyrus. The gray dots represent the two recordings in which the alteration between the zones was detected.

We performed the whole-cell patch-clamp recordings from the principal neurons of the superficial cortical layers in rat brain slices. The recordings were made from slices obtained from both sham-operated rats and rats with freeze-induced lesions. In the latter case, the neurons were located outside the lesion (Figure 1c). No spontaneous epileptiform activity was observed in slices, containing the freeze-induced lesion (such activity was detected only once in 38 slices and was not included in analysis). The slices were perfused with modified ACSF containing a reduced concentration of Mg^2+^ (0.25 mM) and the GABAa receptor antagonist gabazine (10 µM), which led to the epileptiform activity within 2–4 min of perfusion (Figure 7a). In almost all recordings, relatively short discharges of about one-second duration are generated. The overall time course of the discharges was mostly standard in both groups (Figure 7b): following a few Aps, the membrane voltage transitioned to the plateau potential, during which the depolarization block of AP generation was observed. A slow afterdepolarization was observed after the cessation of the discharge. However, the epileptiform activity was significantly more intense in the FCD group (Figure 7c): the average discharge frequency was 0.85 ± 0.08 and 1.53 ± 0.21 discharges per minute in the control (*n* = 24) and FCD (*n* = 21) groups, respectively (*p* = 0.005, Welch’s test).

The discharges were more stereotyped in the FCD group. In the control group, a fraction of atypical, prolonged discharges with significantly increased duration was observed in 10 out of 20 recordings (as in Figure 7c, left panel, the discharges with increased duration are indicated with purple color). Such behavior was observed in only 4 out of 23 recordings in the FCD group (*p* < 0.05, Fisher exact test). The median duration of epileptiform discharges was also significantly higher in the control group (Figure 7d, 0.8 (0.5; 1.6) vs. 0.3 (0.2; 0.6) s in control (*n* = 16) and in FCD (*n* = 21), respectively; *p* = 0.001, Mann–Whitney test). However, we did not observe significant differences in the peak amplitudes of glutamatergic synaptic currents during epileptiform discharges in the two groups ( representative voltage-clamp recordings of epileptiform activity are shown in Figure 7e; the typical synaptic currents at −60 mV are extended in the insets with boxs; the median amplitudes were −3.9 (−2.9, −4.6) vs. −3.0 (−2.2, −4.9) nA in the control (*n* = 6) and FCD (*n* = 7) groups, respectively; *p* = 0.35, Mann–Whitney test, the diagram in Figure 7e).

The slow persistent depolarization of cortical neurons (or depolarizing current, when recorded in the voltage-clamp mode, as in Figure 7e (lower trace), marked with red arrow) was also observed in some recordings, which is consistent with cortical spreading depression (CSD) reported in previous studies [16,17]. In our preparation, the CSD led to the ablation of epileptiform activity for at least 10 min (note the prolonged pause in epileptiform discharge generation following an episode of CSD in Figure 7e). In the control group, only one episode of CSD during the perfusion of slices with modified ACSF was detected in a total of 28 recorded (pooled data for both voltage- and current-clamp recordings). In the FCD group, CSD was observed in 9 out of 37 recordings, which is significantly higher (*p* < 0.05, Fisher exact test).

Taken together, the results show that the epileptiform activity induced in cortex slices by gabazine and decreased Mg^2+^ concentration is significantly more intense in the FCD group. Therefore, it can be assumed that the FCD promotes epileptiform synchronization in our model. To investigate this possibility, we performed dual whole-cell recordings from two neurons located at different distances from the freeze-induced lesion (Figure 7f). The first recorded neuron was located outside the lesion as described above, while the second neuron was located inside the lesion in the same layer (the left panel of Figure 7f represents the relative positioning of the recorded neurons). We performed 16 such dual recordings, in 11 of which the epileptiform discharges could be detected 10–40 ms earlier in a neuron inside the lesion, relative to the neuron outside the lesion (we swapped the relative positioning of the lesion for each recording to eliminate the systemic error that can be introduced if there is a preferred direction of discharge propagation along the cortex); the distribution of delays between the discharges is plotted in Figure 7f). In the remaining five recordings, either the discharges were detected outside the lesion (*n* = 3) or the alternation between the recorded neurons was observed (*n* = 2). Thus, the results suggest that the freeze-induced lesion serves as a source of epileptiform discharges in the implemented model.

## 3. Discussion

In the present study, we analyzed the electrophysiological properties of neurons in the zone adjacent to the microgyria created by a cortical focal freeze lesion and evaluated some features of synaptic transmission in this area. The neurons that we studied were located in the region that was close to the microgyrus but had a normal six-layered cortical structure (Figure 1c). Our data show that, although most of the biophysical properties of neurons are unchanged, the summation of excitatory and inhibitory inputs to neurons is enhanced; moreover, the balance of inhibitory and excitatory synaptic currents is shifted toward excitation, making this region more susceptible to seizure generation. In an in vitro model of epileptiform activity, we observed that the onset of ictal activity occurs earlier in the microgyria region and then spreads to neighboring regions. It is noteworthy that in the cortex of rats with FCD, epileptiform activity is more stereotyped than in control animals, and its frequency is 1.5 times higher.

Our findings generally align with prior in vitro studies, which indicated heightened neuronal excitability in the adjacent zone of microgyria [7,12,18]. However, the underlying causes of this phenomenon remain to be fully elucidated. Possible causes may include changes in the biophysical properties of neurons, changes in the number of synaptic connections and the ratio of excitatory and inhibitory synapses, disruption of connections between different layers, and the probability of mediator release.

The biophysical membrane properties of neurons are highly plastic, and their changes have been described under a variety of pathological and physiological conditions. Specifically, in cases of epilepsy or after induced seizures, alterations can be detected that prompt postsynaptic neurons to initiate firing at reduced levels of depolarization [19,20,21]. Prenatal hypoxia has been shown to cause significant changes in the intrinsic membrane properties of cortical and hippocampal neurons in young rats, including increased input resistance and more depolarized membrane potential [22]. It was previously reported that the neurons in the microgyric cortex exhibited action potentials with a smaller amplitude and slower rise time, compared to sham-operated controls, suggesting that neurons within the lesion may maintain some characteristics of immature cells [11]. The same study reports that microgyric cortex neurons also had less steep f-I relationships. The present study revealed no evident differences in membrane properties between neurons from the FCD and control groups. It is noteworthy that the role of changes in intrinsic excitability in the focal freeze lesion model has received minimal attention from researchers [23,24]. Hablitz and Yang demonstrated that HCN1 immunoreactivity is significantly reduced in the apical dendrites of layer 5 pyramidal neurons in the region adjacent to the microgyrus [23]. In subsequent studies, the researchers observed that layer V neurons near the lesion exhibited more negative resting potentials, larger somatic input resistance, less amplitude of depolarizing sag, and higher firing frequency after applying the same depolarization current than neurons in sham-operated animals. These differences between the two groups disappeared after the application of the Ih blocker ZD7288 [24]. In the present study, we did not find similar differences; however, this cannot be a sufficient argument in favor of the complete absence of abnormalities in HCN channel expression without additional studies. One possible explanation for the discrepancy between our results is that we studied the neurons in layers II–III, not in layer V.

It has been suggested that the change in the ratio of excitatory and inhibitory inputs may be due to the absence of layer 4 in the microgyrus, which causes the reorganization of afferent inputs, especially thalamocortical ones. These inputs find corresponding laminar targets in the cortex surrounding the malformation in the paramicrogyral area [25], which was also demonstrated at the morphological level [26]. Consequently, the predominant factor contributing to the hyperexcitability of the area surrounding the malformation is considered to be its hyperinnervation by afferents that have lost their targets within the malformation [14,15,27,28].

Here, we demonstrated the increase in the paired-pulse ratios (PPR) for both glutamatergic and GABAergic synaptic responses of neurons adjacent to the cortical malformation. The short-term synaptic facilitation that we observed is generally understood to be mediated by presynaptic mechanisms, such as the increased vesicle release probability due to the presynaptic Ca^2+^ dynamics and the size of the readily releasable vesicle pool [29,30]. While other mechanisms, like postsynaptic receptor desensitization, may also play a role in some synapses, at the 50 ms interval that we used here, it is unlikely to meaningfully contribute to the observed changes [31]. It can, therefore, be hypothesized that synaptic inputs coming from the microgyrus have a lower probability of mediator release in both excitatory and inhibitory synapses.

It is plausible that substantial reorganizations are characteristic not only of thalamocortical but also of local cortical connections. This is because we did not stimulate from white matter but from 2 to 3 cortical layers, 50–300 μm from the pyramidal neuron. Therefore, we most likely activated local rather than thalamocortical connections. This hypothesis is further substantiated by the previous observation that both spontaneous and evoked IPSCs exhibited significantly augmented amplitudes in layer 5 paramicrogyral cells when compared to the control group. However, this discrepancy in amplitude was not observed following the blockade of glutamatergic transmission or for miniature IPSCs, indicating that it was attributable to excitatory afferent activity driving inhibitory neurons [14]. Another study has also demonstrated that fast-spiking interneurons in a zone adjacent to the malformation receive increased excitatory synaptic input and may provide increased GABAergic output in that area [15].

The generation of epileptiform activity in malformed cortical regions has been the subject of extensive research. Both in vivo and in vitro studies utilizing the freeze lesion model of cortical microgyria show that under baseline conditions, the freeze lesion per se rarely causes abnormal patterns of neuronal activity or spontaneous seizures [12,27,32]. However, when some form of provocation is applied (like extracellular stimulation or application of chemoconvulsants), the malformed cortex generally displays a substantially higher seizure susceptibility [7,11]. Epileptiform activity in response to extracellular stimulation in slices emerges after a latent period during which there is an increase in both sEPSC and mEPSC frequencies [14,15]. Abnormal EEG patterns have been described in malformed cortex following intraperitoneal injection of kainic acid [33]. A decreased threshold for hyperthermia-induced seizures was observed in freeze-lesioned animals [34]. Similarly to the results of the present study, epileptiform activity induced by ACSF with decreased Mg^2+^ concentration was reported to be more frequent in the malformed cortex compared to the control [35]. The same study reported an increased magnitude of epileptiform events, which was not observed in our preparation and may be due to a different model of epileptiform activity. However, no increase in seizure severity was observed in the PTZ model in vivo [36].

As previously shown, it is not the microgyria itself, but rather an adjacent zone that acquires the capacity to generate synchronous abnormal activity that propagates throughout the cortex [7,12,27,37]. Direct electrical stimulation within the microgyri rarely induced abnormal field potentials, and complete separation of the malformation from the slice with a cut did not eliminate hyperexcitability. Using intrinsic optical signal recordings, it was found that the onset of seizure-like events in a 4-aminopyridine model was restricted to the perilesional area, confirming that this region is hyperexcitable and likely to trigger pathological activity [37]. However, the results of the present study suggest that spontaneous epileptiform events, facilitated by GABA_A_ receptor blockade and Mg^2+^ lowering, mostly originate within the microgyrus and propagate to the adjacent cortical region.

The model of the epileptiform activity implemented in the present study is based on the blockade of GABA_A_ receptors; however, alterations in GABAergic inhibition are considered to be one of the major factors contributing to perilesional hyperexcitability. Region-specific alterations of GABA_A_ receptor-mediated synaptic transmission have been reported in several studies. Quantitative in vitro receptor autoradiography has revealed a 40% reduction of GABA_A_ receptors within the microgyrus and a 15–20% reduction in regions adjacent to the microgyrus [13]. Adult rats have been observed to exhibit a widespread reduction in immunoreactivity of most GABA_A_ receptor subunits throughout the cortex in a hemisphere with a freeze-induced lesion, with the most pronounced reduction detected within the malformation [38].

The microgyric cortex exhibited an augmented number of asymmetric (presumably excitatory) synapses, accompanied by a reduced number of symmetric (presumably inhibitory) synapses [36]. The GABA_A_ receptor subunits were also found to be downregulated both in the microgyrus region and the adjacent area in a freeze-lesion model [38]. A transient decline in the number of PV interneurons was observed within the malformed cortex and in adjacent cortical regions at P13-15, but not at P21 [39]. This decline was more pronounced within the microgyrus itself but extended to the areas up to 2 mm adjacent to it, covering the region from which we recorded in the current study. The same study reported a permanent decrease in the density of PV interneurons in the infragranular layers both within and immediately adjacent to the microgyrus. Alterations in chloride transporter expression in the malformed neocortex of model animals and patients, which should lead to an increased Cl^−^ equilibrium potential, have also been reported in animal models and patients [40,41,42,43,44].

Bumetanide, an inhibitor of the NKCC1 chloride exchanger, selectively reduced epileptiform activity in lesion-containing slices (but not in slices from sham-treated control rats). It also shifted the discharge onset site away from the perilesional area, suggesting that NKCC1 activity is more prominent in lesional/perilesional tissue [37]. The results of the present study also demonstrate a significant increase in the Glu/GABA index for burst stimulation from within the microgyrus, confirming the deficit of synaptic inhibition in that zone. Thus, we can hypothesize that the application of gabazine, implemented in the present study, can result in a differential upregulation of network excitability within and outside of the lesion. A complete blockade of GABA_A_ receptor-mediated synaptic transmission may have a greater effect on neurons adjacent to the microgyrus than on neurons within the microgyrus, making regional alterations of GABA_A_ receptor-mediated synaptic transmission less crucial for the initiation of an epileptiform event. It is also possible that some forms of epileptiform activity can be purely mediated by GABAergic interneurons [45] and, thus, can be directly abolished by gabazine.

Upregulation of glutamate receptors has been reported in malformed cortex [33]. Glutamate receptor subunit expression is locally altered at the site of cortical malformation but is almost absent in neighboring regions [46]. Quantitative in vitro receptor autoradiography shows that AMPA receptors are strongly upregulated both within the microgyrus and, to a lesser extent, outside the microgyrus, whereas NMDARs are upregulated exclusively within the microgyrus [13]. Consequently, the combination of GABA_A_ receptor blockade and NMDA receptor upregulation induced by low concentrations of Mg^2+^ could potentially enhance the manifestation of altered excitatory synaptic transmission properties, thereby shifting the epileptogenic focus to the core of the malformation.

This study demonstrates that the cortical malformation resulting from focal freeze lesions serves as a reliable model for investigating the mechanisms underlying the generation of epileptiform activity in microgyria-type FCD. Our findings indicate that the primary pathogenic mechanism stems from an imbalance of inhibition and excitation, triggered by synaptic perturbations rather than by alterations in the biophysical properties of neurons in the vicinity of the microgyria. Consequently, it is plausible that enhancing seizure control may be attainable by modulating synaptic transmission.

## 4. Materials and Methods

### 4.1. Experimental Animals and Focal Freeze Lesion (FFL) Model

Unilateral multiple FFLs were induced in newborn rats during the first 24 h after birth (P0) to induce neocortical microgyria in the left cortical S1 area, using a modification of the method of Dvorak and Feit [6]. Briefly, newborn rat pups were anesthetized by hypothermia: rats were immersed in wet ice (≈0 °C) for approximately 4–5 min until movement and response to tail pinch were absent. A rostro–caudal longitudinal skin incision (≈8 mm) was made with a scalpel. A cylindrical copper rod with a tip diameter of 1 mm, cooled to approximately −50 °C, was used to induce cortical lesions by freezing. The rod was applied to the cranial surface over the left frontoparietal cortex, ≈2 mm posterior to the bregma, and ≈2 mm lateral to the midline, for 8 s. Similarly, 3 additional freezing lesions were induced ≈1 mm apart in a rostro–caudal direction.

For recovery, the rats were placed on a heating pad under infrared light and held until they were actively moving, after which they were returned to their home cages. Control animals underwent sham surgery (exposed to all surgical procedures except freezing).

All experiments were conducted on male Wistar rats at P21–P23 in accordance with the local guidelines for the treatment of laboratory animals and with the approval of the Ethics Committee of the Sechenov Institute of Evolutionary Physiology and Biochemistry. These recommendations fully comply with the standards for animal research established by Russian and international regulations. Every effort was made to minimize pain and discomfort. Rats were maintained under standard conditions at room temperature with free access to food and water.

### 4.2. Slice Preparation

Brain slices were performed as described in detail earlier [47]. Briefly, the rats were decapitated, the brains were quickly removed, the frontal cortex and cerebrum were removed, and the remaining sections were cut into 350-μm-thick horizontal slices using a vibrating microtome Microm HM 650 (Microm International GmbH, Walldorf, Germany). The cutting bath was filled with the artificial cerebrospinal fluid (ACSF) containing (mM) 126 NaCl, 2.5 KCl, 1.25 NaH_2_PO_4_, 1 MgSO_4_, 2 CaCl_2_, 24 NaHCO_3_, 10 D-glucose, aerated with a gas mixture of 95% O_2_ and 5% CO_2_. The slices were then transferred to oxygenated ACSF and incubated for one hour at 35 °C before electrophysiological recording.

### 4.3. Electrophysiological Recordings

Whole-cell patch-clamp recordings were performed in a perfused slice chamber at 30 °C with a solution flow set at 5 mL/min. Neurons approximately 50–300 µm from the lesion center, in layers II-III of normally formed six-layer somatosensory cortex, were visualized using an Axioskop-2FS Plus microscope (Carl Zeiss AG, Oberkochen, Germany) equipped with differential interference contrast optics and a video camera Sanyo VCB-3512P (SANYO Electric Co., Ltd.; Moriguchi, Osaka, Japan). The passive and active membrane properties were studied based on neurons’ responses to the current steps (1.5 s length every 3 s, amplitudes from −100 to +600 pA in 10 pA increments). Signals were registered with the Multiclamp 700B (Molecular Devices, Sunnyvale, CA, USA) patch clamp amplifier, digitized at 12.5 kHz frequency using LIH 8 + 8 (HEKA Elektronik GmbH, Reutlingen, Germany) AD-DA converter and WinWCP 5 (University of Strathclyde, Glasgow, UK) software. No correction for series resistance was applied.

The pyramidal neurons were identified first visually, by their relatively large soma and distinctive pyramidal shape, and then confirmed by their electrophysiological characteristics, as in our previous work [48]. Most pyramidal neurons in layers 2/3 of the neocortex are regular spiking [49,50]. In our neuron sample, all cells exhibited a regular spiking firing pattern, with prominent frequency adaptation within the train (Figure 4).

Synaptic responses were registered in layer II-III pyramidal cells by stimulating the synaptic inputs from nearby cells with bipolar electrodes using the A365 stimulus isolator (World Precision Instruments, Sarasota, FL, USA) in unipolar mode. The stimulus duration was set to 0.1 ms. The stimulating current amplitudes were chosen based on the amplitude and shape of the elicited responses so that they would be monosynaptic, and the first EPSC would have the amplitude of 150–300 pA. The stimulating current amplitudes ranged from 50 to 1000 µA.

The epileptiform activity in temporal cortex slices was induced by applying a modified ACSF with decreased Mg^2+^ concentration and the added GABA_A_ receptor blocker SR-95531 (gabazine, 10 µM, Alamone labs, Jerusalem, Israel). The solution had the following composition (in mM): 125 NaCl, 3.5 KCl, 2 CaCl_2_, 24 NaHCO_3_, 1.25 NaH_2_PO_4_, 0.25 MgSO_4_, 10 dextrose, 0.01 gabazine.

Current-clamp recordings of the membrane voltage of the principal neurons were performed using a potassium gluconate-based pipette solution with the following composition in (mM): 136 K-Gluconate, 10 NaCl, 5 EGTA, 10 HEPES, 4 ATP-Mg, and 0.3 GTP; pH adjusted to 7.25 with KOH.

Voltage-clamp recordings of the synaptic responses of the principal neurons were performed using a cesium methanesulfonate-based pipette solution with the following composition in (mM): 127 CsMeSO_3_, 10 NaCl, 5 EGTA, 10 HEPES, 6 QX314, 4 ATPMg, and 0.3 GTP; pH adjusted to 7.25 with CsOH.

Voltage-clamp recordings of the epileptiform activity were performed at −60 mV using the modified pipette solution with added blockers of the voltage-gated sodium and potassium ion channels: 110 K-Gluconate, 10 Na-Gluconate, 20 Tetraethylammonium chloride, 6 QX314 (Alomone labs), 10 HEPES, 5 EGTA, 4 Mg-ATP, 0.3 Na-GTP; pH adjusted to 7.25 with KOH.

### 4.4. Morphology

In this study, we followed the methodology described in our previous publication for preparing frozen brain sections and performing Nissl staining [51]. The Nissl-stained sections were examined under a light microscope, Leica Microscope AF 7000 (Leica Microsystems, Wetzlar, Germany), at 50× magnification and digitally imaged.

### 4.5. Data Analysis

The electrophysiological data were converted to the ABF format using the Review 5.0.2 software (Bruxton Corp., Seattle, WA, USA) and analyzed using custom software written in the Python 3.13.1 programming language with NumPy 2.2.2 [52], SciPy 1.15.1 [53], and pyqtgraph 0.13.7 [54] modules.

The RMP was measured as the mean of the voltage trace at the zero input current. The R_i_ was estimated as the slope of the linear regression for the current–voltage relationship for the subthreshold steps. The membrane τ was calculated as a parameter of a single exponential function fitted to the beginning of the voltage response to a hyperpolarizing current step (20 pA). The hump amplitude was calculated as the voltage peak at the start of the depolarizing current step 10 pA below the rheobase, relative to the steady-state voltage at the end of the same current step for each neuron.

We analyzed the properties of the APs at the rheobase current. The voltage threshold was determined as the point at which the first derivative of the voltage (dV/dt) exceeded 5 mV/ms. The amplitude was determined as the peak AP voltage relative to its threshold. The half-width was determined as the AP width at the voltage level of its half-amplitude, relative to a threshold. The fAHP peak was determined as the point at which the voltage decay markedly slowed down below 5 mV/ms. The mAHP peak was measured as the lowest value that the voltage reaches after the AP peak relative to its threshold. The mAHP latency was measured between the fAHP and mAHP peaks.

The rheobase current was determined as the minimal current sufficient to induce the AP generation. The time of the first spike was measured from the start of the current step to the time of the first AP threshold. For each neuron, we plotted the CFC (AP generation frequency in relation to the amplitude of the depolarizing current injected into the cell) and measured such parameters as the rheobase (minimal current amplitude sufficient to evoke the AP), maximal AP frequency before the depolarizing block of the voltage-gated Na^+^ channels occurs, leading to the downward slope of the end of the CFC, and the maximal slope of the CFC.

The current eliciting the maximal AP frequency was determined as the minimal current sufficient to elicit the AP generation with the maximal frequency. The early frequency adaptation was determined as the ratio of the second interspike interval to the first one, and the late adaptation—as the ratio of the last interspike interval to the first one on the next current step after the rheobase (rheobase + 10 pA).

We analyzed the epileptiform activity that occurred during the 10 min time interval starting 20 min after the application of a modified ACSF. The recordings in which cortical spreading depression (CSD) was detected during this period were not used for the estimation of discharge frequency and duration. The average epileptiform discharge duration was estimated in current-clamp mode at a −45 mV level.

### 4.6. Statistical Analysis

Statistical analysis was performed using Python with Pandas, NumPy, and SciPy modules and visualized using the Matplotlib 3.10.0 module [55]. Data were filtered for outliers using the Iglewicz and Hoaglin test with the threshold set at 3.5. A total of 0 to 3 points was excluded from each dataset. Multiple group comparisons were performed using one-way ANOVA with Tukey’s HSD post hoc test. The percentage of the cells with spike doublets in post-SE groups was compared with control using the two-sided Z-test for proportions. All data are represented as mean ± standard error.

No statistical method was used to predetermine the sample size. The sample sizes were estimated based on our previous studies for similar types of electrophysiological and morphological studies. The number of electrophysiological recordings per animal was determined by the length of the experiment; for synaptic responses, it was 2 on average, while for action potentials, it was 8–9.

## Data Availability

The data presented in this study are available on request from the corresponding author.
